# A rare case of pituitary stalk interruption syndrome (PSIS) presenting as short stature in an 8‐year‐old female

**DOI:** 10.1002/ccr3.9274

**Published:** 2024-08-05

**Authors:** Kamana Sen, Kritick Bhandari, Suman Simkhada, Karuna Humagain, Prasnna Basnet, Pawan Kumar Shah

**Affiliations:** ^1^ KIST Medical College and Teaching Hospital Lalitpur Nepal; ^2^ Department of Endocrinology KIST Medical College and Teaching Hospital Lalitpur Nepal

**Keywords:** congenital anomaly, endocrinopathy, pituitary stalk interruption syndrome, short stature

## Abstract

Pituitary stalk interruption syndrome is a rare, congenital abnormality. Early identification and treatment can improve patient prognosis and quality of life and prevent adverse effect on growth and development. The patient described is an 8‐year‐old child with a history of short stature.

## Graphical abstract

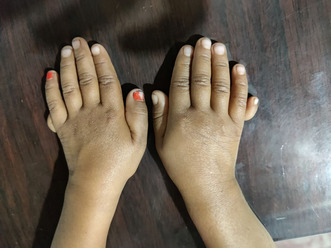

## 1 INTRODUCTION

The absence or thinning of the pituitary stalk, absent or ectopic posterior pituitary, and anterior pituitary hypoplasia or aplasia constitute the classic triad of anatomical variation associated with pituitary stalk interruption syndrome (PSIS).^1^ PSIS is a rare congenital syndrome. The exact prevalence of PSIS remains unknown.^2^ To date, the etiopathogenesis of PSIS remains elusive. However, several theories, such as impaired organ development during the embryonic stage brought on by genetic and/or environmental factors, and the role of perinatal injuries have been proposed.^3^ PSIS may manifest as either an isolated deficiency of growth hormone or there may be underproduction of multiple anterior pituitary hormones.^3^ Posterior pituitary function usually remains unaffected.^4^ Clinically, it can have variable presentations such as hypoglycemia in newborns, neonatal hypothyroidism, cryptorchidism or micropenis in boys, late or no attainment of pubertal changes, and/or short stature.^4^ Diagnosis depends upon clinical evaluation, endocrinological reports, and imaging with MRI being the confirmative test.^5^ The mainstay of treatment is hormonal replacement therapy, which involves lifelong hormone substitution and long‐term follow‐up.^6^

## 2 CASE HISTORY

An 8‐year‐old girl presented with a complaint of short stature to our outpatient department of Pediatrics and Endocrinology. According to her parents, she had always been shorter in stature compared to her classmates or children of her age. Her birth history was uneventful. The patient was born via normal delivery at full term without any neonatal intensive care unit (NICU) stay. Her vaccination history was up‐to‐date according to the National Immunization Schedule. There was no history of developmental defects, and currently, she is enrolled in her age‐appropriate grade at school and is doing well in her studies. She maintains cordial relationships with her friends, family members, and relatives; her social activities are appropriate for her age. There was no history of chronic disease, surgical intervention, hospitalization, or similar presentations in her family members or relatives.

**FIGURE 1:**
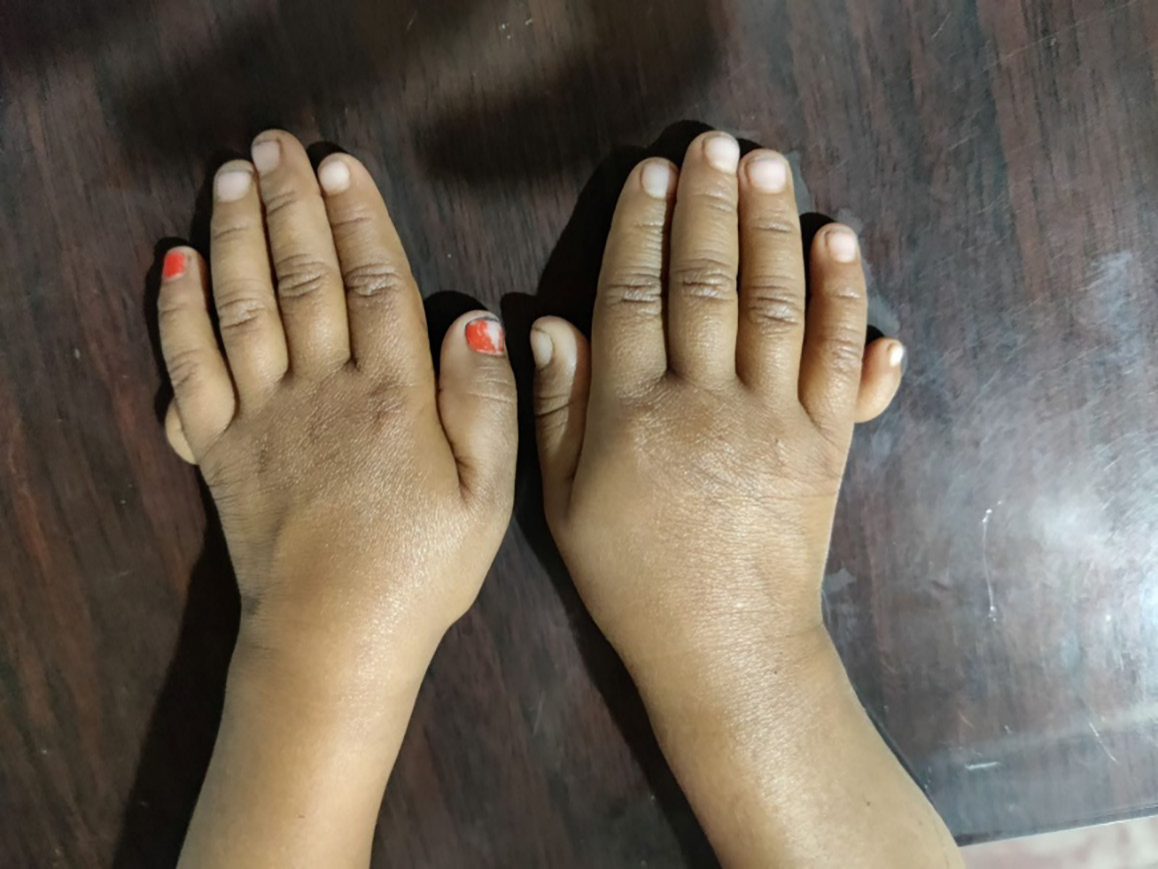
Bilateral polydactyly with an extra digit outgrowing from both the 5th digits.

During analysis of the anthropometric parameters, the patient was found to have not attained adequate weight and height for her age (Table 1). Anthropometric analysis was performed using WHO child growth standards. During the clinical assessment, there were no facial or genital abnormalities. Her neurological examinations did not reveal any defects. The rest of her systemic examinations were also found to be normal. The patient had bilateral hand polydactyly with one more digit attached to each of her fifth fingers (Figure 1).

**TABLE 1:** Anthropometric parameters of the patient.

Anthropometric parameters	Values
Weight	10 kg
Height	88 cm
BMI	12.9 kg/m^2^
Weight for age	< 3rd percentile
Height for age	< 3rd percentile
BMI for age	< 3rd percentile
Head circumference	46 cm
Abdominal girth	43 cm
Upper segment/lower segment (US/LS)	1.1 (47 cm:41 cm)

Abbreviation: BMI, body mass index.

## 3 METHODS

Laboratory investigations revealed a normal complete blood count (CBC), peripheral blood smear, serology panel, liver function test, renal function test, and urinalysis. As part of the investigations, 25‐Hydroxy vitamin D, Serum calcium, and hormonal analysis were performed, and the results were as follows (Table 2):

**TABLE 2:** Relevant laboratory reports of the patient.

Tests	Result	Unit	Reference range
Free triiodothyronine (FT3)	2.79	pg/mL	2.0–4.2
Free thyroxine (FT4)	0.89	ng/dl	0.89–1.72
TSH, ultrasensitive	4.18	μIU/mL	0.3–4.5
Human growth hormone (hGH)	0.35	ng/mL	0.120–7.79
Human growth hormone (hGH) after 1 h of levodopa stimulation test	0.37	ng/mL	0.120–7.79
Insulin like growth factor‐1 (IGF‐1)	**36.1**	ng/mL	60.0–350.0
Luteinizing hormone	**1.00**	mIU/mL	Follicular phase:2.95–13.65
			Ovulation phase:13.65–95.75
			Luteal phase:1.25–11.00
Follicle‐stimulating hormone	**1.21**	mIU/mL	Follicular phase: 4.46–12.43
			Ovulation phase:4.88–20.96
			Luteal phase:1.96–7.70
Fasting morning cortisol	8.9	μg/dL	Normal: 6.4–22.8 μg/dL
25‐Hydroxy vitamin D	27.5	ng/mL	Deficiency<20.0
			Insufficiency 20–30
			Sufficiency 30–100
			Toxicity>100
Serum calcium	8.30	mg/dl	8.1–10.5

*Note*: An x‐ray of the left hand was performed, which revealed that the bone age was 4–5 years according to the Greulich and Pyle method while the patient's chronological age was 8 years. The bold values indicate levels either above or below the normal reference range (abnormal lab results).

### 3.1 MRI brain findings

**FIGURE 2:**
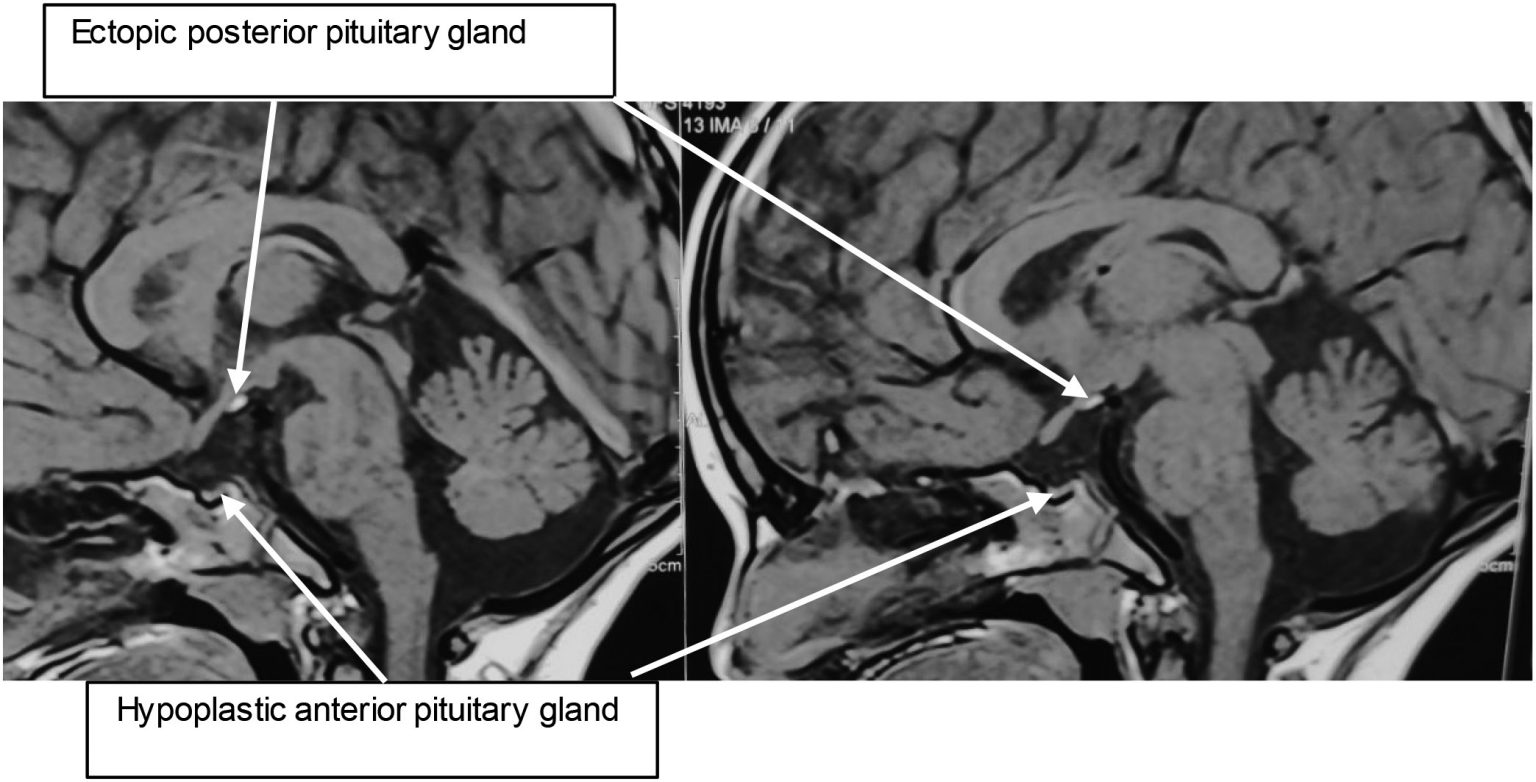
Sagittal T1 weighted MRI with the classic triad—pituitary hypoplasia, absent stalk and ectopic posterior pituitary. The pituitary gland is relatively small in size and measures 4.1 × 3.6 × 1.08 mm (TR × AP × CC). No definite nodule could be identified in plain or dynamic scans. Tl bright posterior pituitary gland is not visualized in the pituitary fossa but is seen posterior to the base of the infundibulum and inferior to hypothalamus.

**FIGURE 3:**
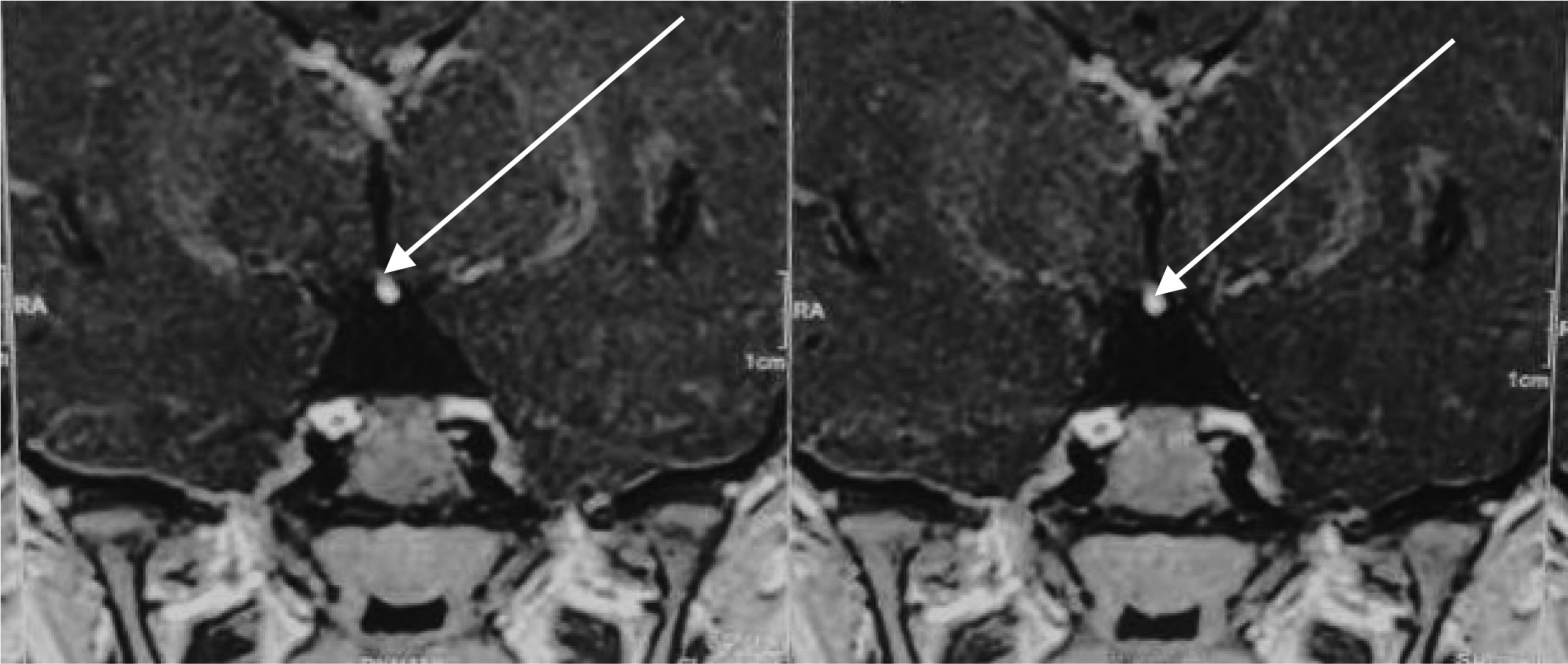
Coronal post‐contrast magnetic resonance imaging of the brain shows an enhancing nodule at the inferior aspect of the optic chiasma (white arrow), suggestive of an ectopic posterior pituitary gland.

MRI of the brain revealed a hypoplastic anterior pituitary gland, thin pituitary stalk, and ectopic posterior pituitary located posterior to the base of the infundibulum and inferior to the hypothalamus. There were no other associated abnormal brain findings. The pituitary gland was relatively small measuring 4.1 × 3.6 × 1.08 mm. No definite nodule could be identified on plain or dynamic scans. On T1, the bright posterior pituitary gland was not visualized in the pituitary fossa but was observed posterior to the base of the infundibulum and inferior to the hypothalamus. A thin pituitary stalk was noted (Figure 2). The size, position, and configuration of the sella were normal. The brain morphology was normal with normal parenchymal signal intensity. There was no evidence of brain edema, recent hemorrhage, mass effect, or infarction. Coronal post‐contrast magnetic resonance imaging of the brain showed an enhancing nodule at the inferior aspect of the optic chiasma (white arrow), suggestive of an ectopic posterior pituitary gland (Figure 3).

The diagnosis of PSIS was confirmed after a thorough evaluation of her clinical history and examinations, and laboratory and radiological investigations. The patient's parents were informed about the diagnosis, counseled about the importance of early hormonal replacement therapy and treatment, and provided a detailed explanation of the patient's prognosis. However, due to financial constraints, they denied treatment and the patient was lost to follow‐up.

## 4 DISCUSSION

One of the rare etiologies of hormonal dwarfism is PSIS. PSIS is a congenital pituitary anatomical defect that is considered very rare. It was first reported by in 1987, in which they demonstrated pituitary stalk transection and formation of the ectopic lobe on MRI in 10 patients.^7^ This was evident as a high‐intensity area present in an ectopic location.^7^ Anatomically, it is characterized by a specific triad, an absence or hypoplasia of the anterior pituitary gland, thinning or absence of the pituitary infundibulum, and an ectopic location of the posterior pituitary.^1^ The epidemiological distribution of PSIS varies among the literature, with an average age at presentation ranging from as low as 4.8 ± 2.7 years^8^ to as high as 19.7 ± 6.7 years.^9^ Most research also demonstrates that PSIS is more predominant in males than females with the percentage of males ranging from 64% to 87%.^8^, ^9^, ^10^ Its incidence is not clear but it has been estimated that approximately 7% of cases of nonacquired GH deficiency are due to PSIS.^11^

The etiology of PSIS is still unclear. The development of PSIS may depend upon perinatal adverse events, as PSIS has been reported in several deliveries complicated by breech delivery, hypoxia, and dystocia.^11^ A study by Fernandez‐Rodriguez et al.^12^ also demonstrated that 26.9% of patients with PSIS have a traumatic birth history or adverse events during the perinatal period. It has been postulated that stretching of the stalk between the pituitary gland and the brain during head trauma associated with breech delivery causes mechanical rupture of the stalk.^13^ However, this assumption has been viewed from an opposite perspective in several studies, which hypothesize that pituitary abnormalities may, in turn, lead to breech delivery and neonatal hypoxemia rather than being the cause.^13^, ^14^ It has been suggested that congenital developmental abnormalities of midline structures lead to the failure of the descent of the neurohypophysis into the sella turcica and, as a result, anterior hormone secretion decreases leading to increased breech presentation.^13^ Genetic mutations could also be the likely cause of PSIS, as reported by some studies, and the fact that 48% of those with PSIS also present with extra‐pituitary malformations also hints at the role of such mutations in its pathogenesis.^15^ These genetic mutations may include but are not limited to, *HESX1*, *OTX2*, *SOX3, LHX4*, *PROP, PROKR2*, *CDON*, holoprosencephaly‐related genes *TGIF* and *SHH, GPR161* and *ROBO1*.^11^ However, these gene mutations are reported in less than 5% of patients with PSIS, suggesting that most of the genes involved have yet to be discovered.^11^

PSIS can present in a non‐specific pattern, the presentation of which depends upon whether there is an isolated growth hormone deficiency or multiple systemic involvement due to multiple hormone deficiencies.^15^ In children presenting with complaints of growth retardation and delayed puberty, PSIS should always be considered an important differential diagnosis. Features suggestive of neonatal hormonal deficiency may also be the initial presentation, such as low blood glucose level, jaundice, and/or genital defects such as failure of descent of testes into the scrotum in males.^16^ Usually, these patients lag in terms of sexual development.^16^ However, PSIS can also present atypically, with Lee et al.^17^ reporting a patient with multiple hormonal deficiencies without growth alterations, and another patient was diagnosed with PSIS who initially presented with recurrent seizures due to hyponatremia as a result of ACTH deficiency.^18^ The loss of the septum pellucidum, malformations relating to the central nervous system and/or craniofacial abnormalities are some common extra‐pituitary manifestations associated with PSIS.^11^ However, our patient had no such craniofacial malformations, but, she presented with bilateral polydactyly with an extra 6th digit on both her hands.

The diagnosis of PSIS is often delayed because it is difficult to timely validate its clinical findings, such as decreasing growth velocity. Careful evaluation of the clinical features, endocrinological investigation results, and suggestive findings on contrast‐enhanced MRI usually lead to the diagnosis.^5^ MRI is the cornerstone for the diagnosis of PSIS. MRI findings constitute a triad of an absent pituitary stalk, an absence of the adenohypophysis, and an ectopic posterior pituitary. Variations in MRI findings can include the location of the posterior pituitary gland, the height of the anterior pituitary which may range from being absent to being of normal length, or stalk variations (thin, normal, or absent). Sometimes an ectopic posterior pituitary may be the only abnormality.^2^

Prompt and appropriate hormone substitution therapy remains the cornerstone of treatment for all patients with PSIS. Patients may receive a combination of GH, levothyroxine, cortisol, testosterone or estrogen, and desmopressin. This combination therapy depends on the specific hormone deficiencies present in the patient. Notably, the hormone profile at diagnosis is not stable, and long‐term follow‐up is imperative.^19^ Bar et al.^16^ also recommend regular follow‐up of children with growth retardation as crucial for early diagnosis of PSIS, as they found in their study that decreased growth velocity was present 2–3 years before referral to the higher center.

## 5 CONCLUSION

PSIS is an uncommon congenital abnormality with a relatively vague etiology. It is important to diagnose it as soon as possible to initiate early hormone replacement and follow‐up. Such prompt treatment may ensure a normal or near‐normal maturation in adult height and secondary sexual characteristics. Since these endocrinological abnormalities are difficult to treat and difficult to explain to patients, a strong doctor‐patient relationship is as important as pharmacological treatment. As PSIS can impose several barriers to patients in attaining a normal lifestyle, a multidisciplinary approach with long‐term follow‐up is a necessity for the proper management of the patients.

## AUTHOR CONTRIBUTIONS

**Kamana Sen:** Conceptualization; data curation; methodology; writing – original draft; writing – review and editing. **Kritick Bhandari:** Conceptualization; data curation; methodology; writing – original draft; writing – review and editing. **Suman Simkhada:** Data curation; investigation; methodology; supervision; validation; writing – review and editing. **Karuna Humagain:** Writing – review and editing. **Prasnna Basnet:** Writing – review and editing. **Pawan Kumar Shah:** Writing – review and editing.

## FUNDING INFORMATION

No funding was received to assist with the preparation of this manuscript.

## CONFLICT OF INTEREST STATEMENT

The authors declare that they have no known competing financial interests or personal relationships that could have appeared to influence the work reported in this paper.

## CONSENT

Written informed consent has been obtained from the patient to publish this report in accordance with the journal's patient consent policy.

## Data Availability

Any data used in the manuscript can be made available if asked upon by the chief editor.
